# A Rare Presentation of Intraosseous Stab Wound by Knife in the Left Forearm

**DOI:** 10.7759/cureus.85830

**Published:** 2025-06-12

**Authors:** Salim Al Lahham, Rana Farsakoury, Abdulqadir J Nashwan

**Affiliations:** 1 Plastic and Reconstructive Surgery, Hamad Medical Corporation, Doha, QAT; 2 Nursing and Midwifery Research, Hamad Medical Corporation, Doha, QAT

**Keywords:** forearm, intraosseous, penetrating injury, plastic and reconstructive surgery, stab wound

## Abstract

The patient in this case report is a 17-year-old male individual who presented as a victim of an assault by knife attack that resulted in a cut below the left elbow, and the knife was stuck at the site of injury. He complained of pain at the site of injury, in addition to an inability to pull out the knife. Physical examination showed a knife stuck below the elbow with normal sensation, pulsations, and range of motion. Laboratory investigations and an X-ray were ordered for the patient. X-ray showed a penetrating knife wound through the left forearm, passing through the radius and ulna, with swelling of the overlying soft tissues of the forearm. The patient was shifted to the operating theater for limb-saving surgery. Intraoperative exploration showed a penetrating wound by a knife through the left forearm, passing through the radius and injuring one of the venae comitantes of the ulnar artery and some perforators. The knife was removed, and an X-ray was taken to check for a fracture of the radius, which showed only a through-and-through tract with no obvious fracture. Hemostasis was achieved, and the wound was closed. The patient was discharged with regular follow-up appointments.

## Introduction

Stabbing is one of the most common methods of homicide worldwide, yet intraosseous knife injuries remain exceedingly rare [1]. A complicated interaction between the sharpness of the knife, the region of the body, and alignment with cleavage lines of the skin, the angle of attack, and the relative movement of the person stabbing about the victim being stabbed determines the force needed to enter the skin and body [1]. When determining how sharp a tool is, especially a knife, some degree of quantification is more manageable [1]. In this article, we describe a rare case of a knife wound that penetrated the left forearm and passed through the radius. It was treated surgically, and the final aesthetic and functional results were good. Our goal was to educate surgeons about the numerous ways that stab wounds might manifest themselves and to provide them with tips for handling similar situations.

## Case presentation

**Figure 1:**
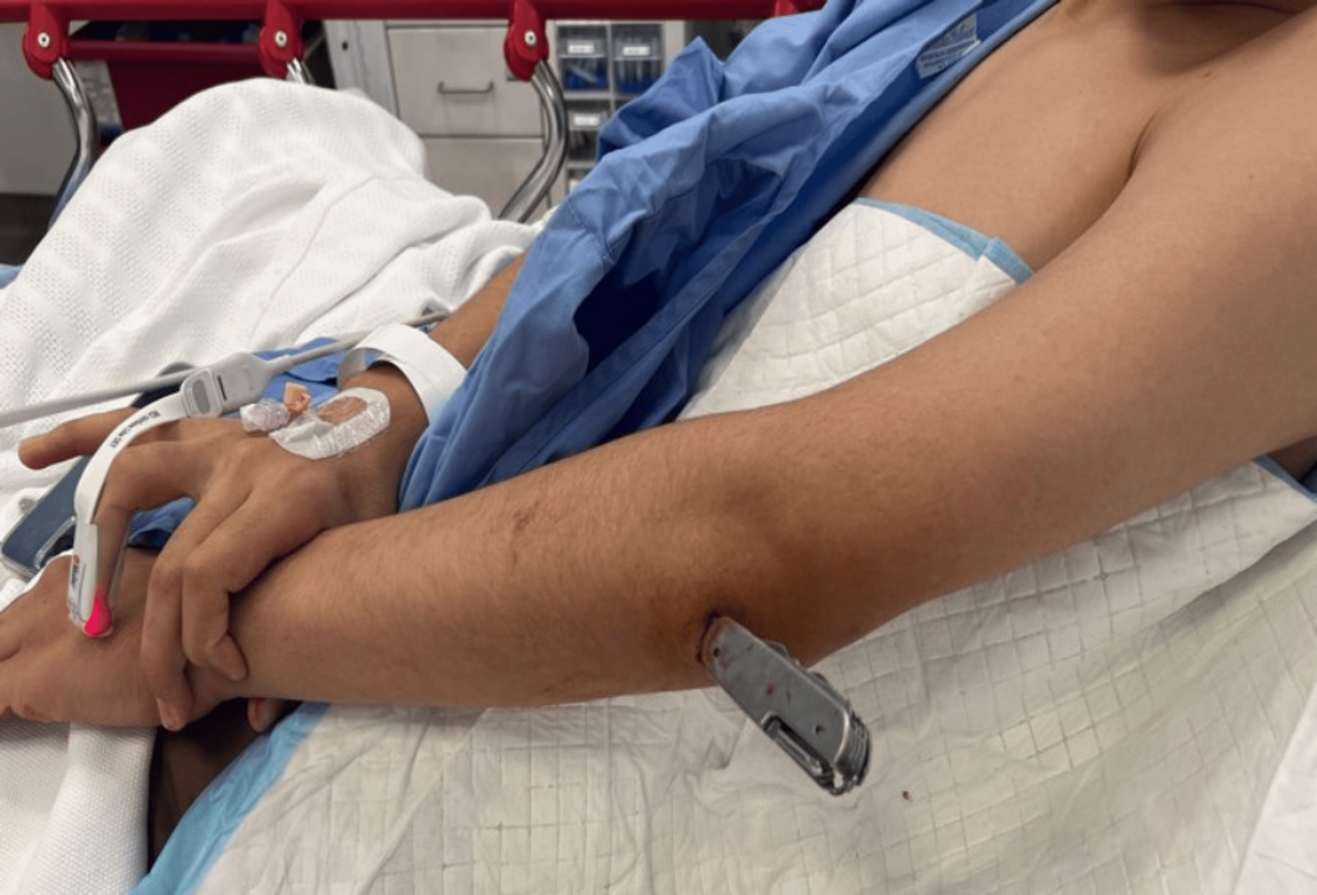
Preoperative photograph of the patient’s left forearm showing the penetrating knife injury.

A 17-year-old male individual presented to the emergency department after being assaulted with a knife, resulting in a penetrating injury below the left elbow with the knife still lodged at the site (Figure 1).

The patient reported that the knife was thrown from a 4-meter distance while he was attempting to protect himself with his left arm. His medical history was unremarkable except for a tonsillectomy. He complained of pain and an inability to remove the knife despite multiple attempts. On examination, the knife was lodged dorsally 2 cm below the elbow. Sensation, pulsations, and range of motion were normal, and the volar skin remained intact. Doppler assessment showed normal perfusion, and there were no clinical signs of ischemia or vascular compromise, so CT angiography was not indicated.

**Figure 2:**
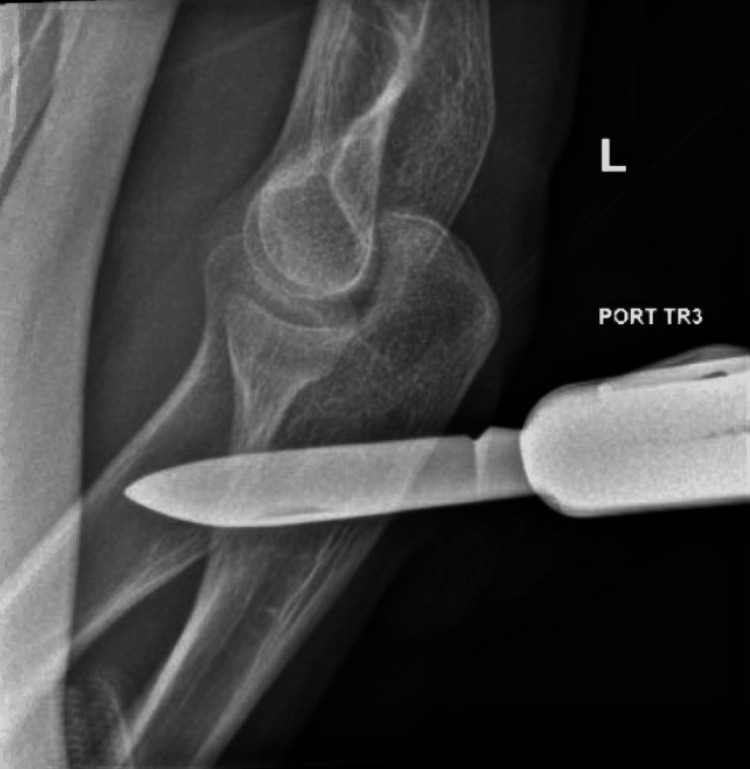
X-ray image of the patient’s left forearm. X-ray image of the patient’s left forearm, showing the penetrating knife through the left forearm, passing through the radius/ulna with swelling of the overlying soft tissues of the forearm.

An X-ray of the left forearm showed a penetrating knife injury through the radius and ulna with overlying soft tissue swelling (Figure 2).

The differential diagnosis included vascular injury, nerve damage, and potential fractures. Based on the clinical findings and imaging results, the patient was scheduled for limb-saving surgical intervention to prevent further damage.

**Figure 3:**
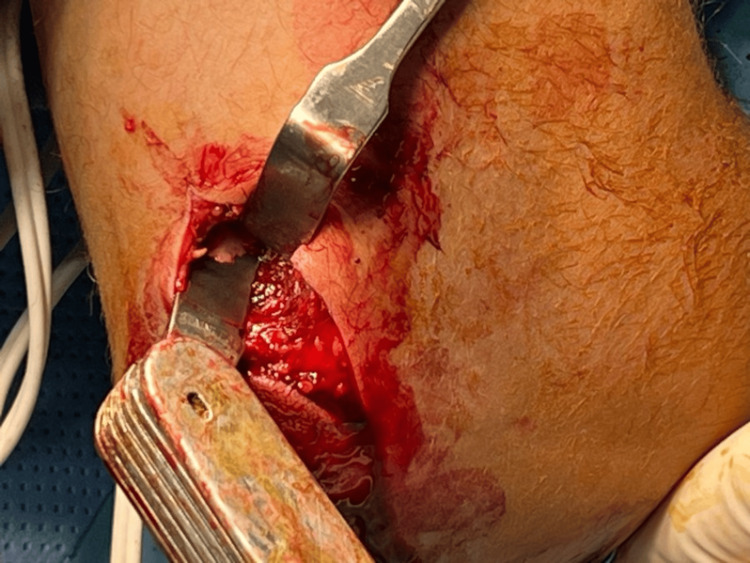
Intraoperative photograph of the patient’s left forearm. Intraoperative photograph of the patient’s left forearm, showing the knife stuck inside the radius.

Intraoperative exploration revealed a penetrating wound through the radius with injury completely cut to one of the venae comitantes of the ulnar artery and some perforators, which were cauterized (Figure 3). No injury to the surrounding muscles or tendons was noted.

**Figure 4:**
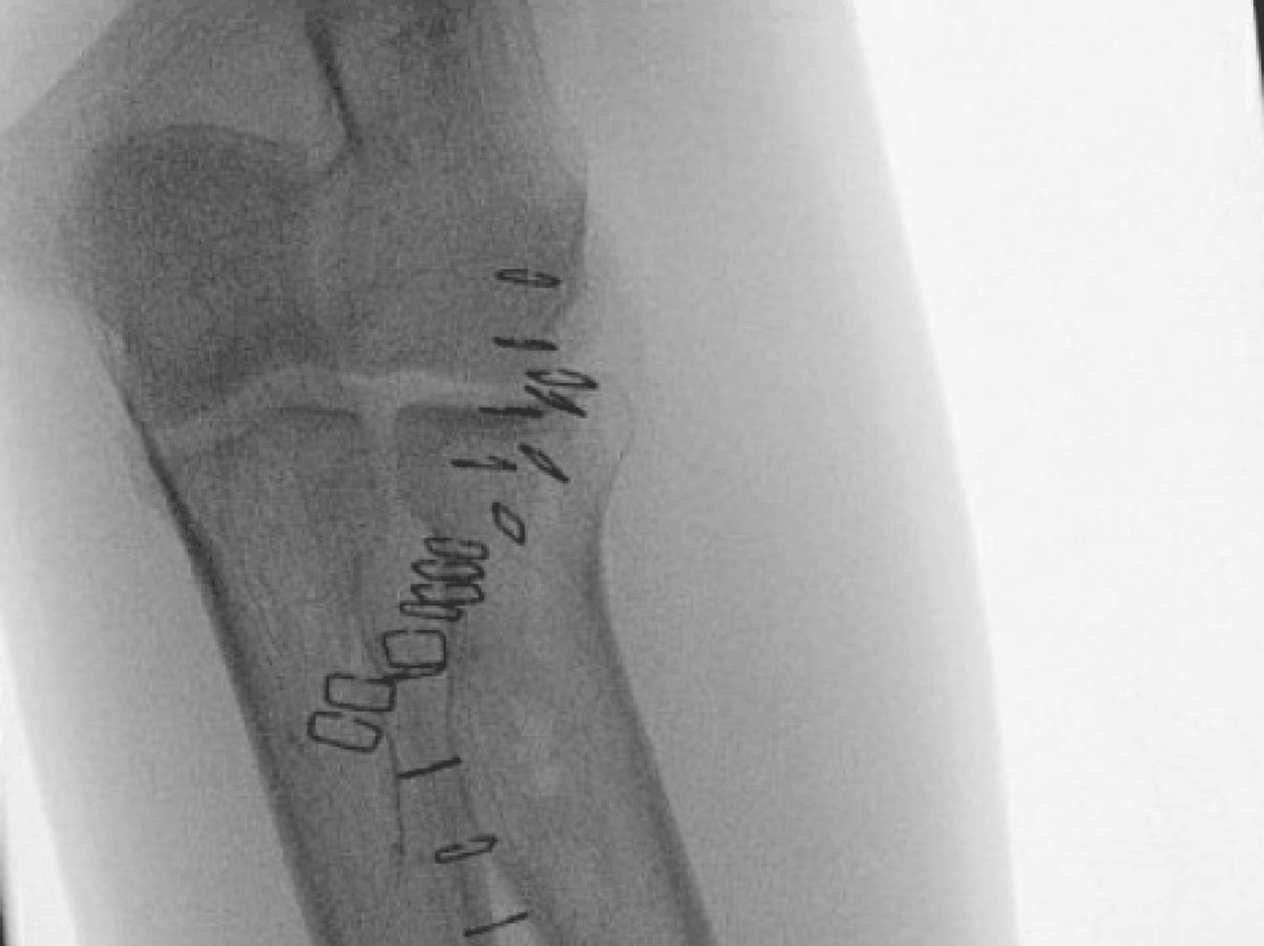
X-ray image of the patient’s left forearm. X-ray image of the patient’s left forearm, showing through-and-through tract in the radius with no obvious fracture.

A volar incision was made to access and evaluate the vascular structures, which was not possible through the dorsal entry point. Due to the urgent nature of the case, volar intraoperative photography was limited. The knife was removed. An X-ray confirmed no fracture but showed a through-and-through tract (Figure 4).

Hemostasis was achieved, and the wound was closed in layers using staples. The forearm was immobilized with an above-elbow slab.

**Figure 5:**
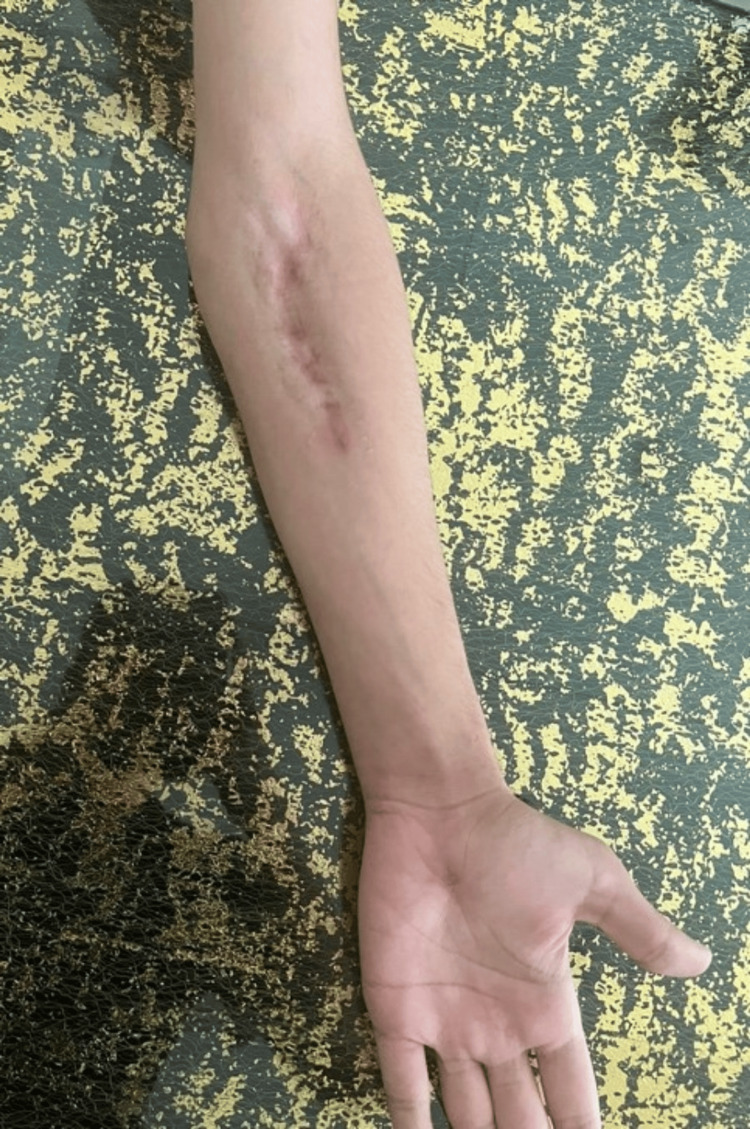
Postoperative photograph of the patient's left forearm, showing the outcome of the operated region.

Nineteen days postoperatively, the patient reported significant improvement in the quality of life and was satisfied with the outcome. He was followed up at 2 and 4 months, showing full active elbow flexion (0-140°), wrist extension (0-70°), and forearm rotation (pronation and supination 0-85° each) without pain. He also had intact pain and temperature sensation. A hypertrophic scar was noted, which improved with two triamcinolone injections (Figure 5).

## Discussion

Knife retention from an intraosseous stab wound is extremely uncommon because it takes a lot of force to impale a sharp object through bone. We think the prevalence of nonfatal stabbings is higher than what has been noted in the literature, as in the case of our patient. According to studies conducted to aid in the production of body armor, the maximum energy in a stabbing motion is roughly 115 J, and the load created by the knife when it makes contact with the target is approximately 1000 N [2,3]. The entry site of the knife blade into the patient's metaphysis most certainly led to the possibility of bone damage.

For the best possible outcome, physicians must conduct a thorough, prompt, and accurate assessment of all injured extremities, evaluating vascular, nerve, and muscular damage [4]. According to the literature on penetrating injuries of the upper extremities, neurovascular impairment and bone penetration have been reported [5,6]. A multidisciplinary team should oversee the management of this type of injury to ensure proper care in the event of a vascular or neurological injury [7]. However, our case did not involve any neurovascular deficits or forearm fractures.

Despite certain examples documented in the literature [5,8-10], the treatment we recommended was in line with the recommendations for any retained foreign body [11-13]. Abboud et al. reported a case of a 53-year-old woman who sustained a stab wound to her left arm, with the knife blade lodged in the proximal metaphyseal region of the left humerus. The foreign body was successfully removed using pliers [5]. Similarly, Quah et al. described a 23-year-old man with a stab wound to the left arm, where the blade had fully penetrated and become embedded in the humerus, requiring osteotomy for its removal [9]. Vide et al. presented a case in which a blade fragment was lodged intraosseously and extended into the joint [8]. The foreign body was removed through a posterior shoulder approach, carefully selected based on the injury’s anatomical location [8]. Likewise, Baghbani et al. described a case of a stab wound to the right shoulder with penetration into the scapula [10]. The patient was successfully managed using a posterior approach, with a multidisciplinary team of orthopedic surgeons and a standby vascular surgeon [10]. All patients had favorable outcomes, achieving full functional recovery at follow-up.

## Conclusions

In our patient, the foreign body was removed in the operating theatre under controlled circumstances after primary and secondary surveys of this trauma patient were completed, since it might indicate a sizable vascular injury that needs to be repaired or cause hemodynamic instability. Debridement and extensive irrigation should be done once the foreign body has been removed. The current case report offers a hint for how to treat retained intraosseous stab wounds because there aren't enough reports of them in the literature. It is also important to individualize the management in similar trauma cases.
